# Self-Alignment MEMS IMU Method Based on the Rotation Modulation Technique on a Swing Base

**DOI:** 10.3390/s18041178

**Published:** 2018-04-12

**Authors:** Haifeng Xing, Zhiyong Chen, Haotian Yang, Chengbin Wang, Zhihui Lin, Meifeng Guo

**Affiliations:** Engineering Research Center for Navigation Technology, Department of Precision Instruments, Tsinghua University, Beijing 100084, China; xhf15@mails.tsinghua.edu.cn (H.X.); chendelta@mail.tsinghua.edu.cn (Z.C.); yang-ht17@mails.tsinghua.edu.cn (H.Y.); wcb17@mails.tsinghua.edu.cn (C.W.); linzh15@mails.tsinghua.edu.cn (Z.L.)

**Keywords:** MEMS IMU, rotation modulation technique, swing base, inertial frame-based alignment, strong tracking filter

## Abstract

The micro-electro-mechanical-system (MEMS) inertial measurement unit (IMU) has been widely used in the field of inertial navigation due to its small size, low cost, and light weight, but aligning MEMS IMUs remains a challenge for researchers. MEMS IMUs have been conventionally aligned on a static base, requiring other sensors, such as magnetometers or satellites, to provide auxiliary information, which limits its application range to some extent. Therefore, improving the alignment accuracy of MEMS IMU as much as possible under swing conditions is of considerable value. This paper proposes an alignment method based on the rotation modulation technique (RMT), which is completely self-aligned, unlike the existing alignment techniques. The effect of the inertial sensor errors is mitigated by rotating the IMU. Then, inertial frame-based alignment using the rotation modulation technique (RMT-IFBA) achieved coarse alignment on the swing base. The strong tracking filter (STF) further improved the alignment accuracy. The performance of the proposed method was validated with a physical experiment, and the results of the alignment showed that the standard deviations of pitch, roll, and heading angle were 0.0140°, 0.0097°, and 0.91°, respectively, which verified the practicality and efficacy of the proposed method for the self-alignment of the MEMS IMU on a swing base.

## 1. Introduction

Inertial navigation systems (INSs) based on high precision inertial gyros tend to be bulky and costly, which limits their application. With the technical development of micro-electro-mechanical-systems (MEMSs), MEMS-based inertial sensors have achieved success in commercial fields such as pedestrian navigation, robotics, unmanned aerial vehicles (UAVs), and autonomous underwater vehicles (AUVs) because of their many attractive features such as being low cost, small, and lightweight, with lower power consumption [1,2]. However, current MEMS inertial sensors have lower accuracy than high end inertial measurement units (IMUs). To improve the navigation accuracy of MEMS INS, the accuracy of MEMS inertial sensors can be further improved by developing the manufacturing technology, but this requires high cost investments and long-term technological improvements [3]. Another way to improve navigation accuracy is using the rotating modulation technique (RMT), which is an error compensation technique. When the MEMS inertial sensor accuracy is determined, the introduction of this technique can effectively mitigate the constant error of the MEMS inertial sensors [4,5].

Determining the initial values of the attitude angles, which is the initial alignment, is essential prior to starting navigation [6,7]. More specifically, the alignment determines the initial attitude relationship between the body frame and the navigation frame, meaning the transformation matrix from the body frame to navigation frame must be solved. For rotary INSs, the alignment determines the attitude relationship between the IMU frame and the navigation frame, and then the transformation matrix from the body frame to the navigation frame can be calculated according to the rotation angle. Only when the alignment is completed can the horizontal attitude and heading angle be obtained, and then navigation can be performed. Therefore, the alignment is the prerequisite for the INS to determine attitude and navigate. High end INSs can self-align without other sensors in a less disturbed environment by applying conventional analytical coarse alignment and using the Kalman filter for fine alignment [8,9]. However, static-base alignment has some limitations. When the INS has to be aligned when on a swing base, the measurement of the rate of earth rotation and gravity information are seriously affected. A swing condition means that the base of the INS is not fixed; instead, it exists in a disturbing circumstance, such as a ship during mooring, or a vehicle affected by a gust of wind or engine vibration. In the swing condition, the base generally does not move or the velocity changes very slowly, but the attitude changes more obviously. Since the earth’s rotation rate is too small, and the magnitude of the angular rate caused by the disturbance may be several orders higher than the rotation rate of the earth [10], the conventional analytic alignment method cannot be used. As such, researchers are focusing on alignment under swing base conditions by separating the disturbance information [11,12,13]. Under swing conditions, the inertial frame-based alignment (IFBA) algorithm is often used. This algorithm introduces a body inertial frame and uses the earth’s rotation rate, alignment time, and the direction change of the local gravity in inertial space to calculate the north of the earth [14,15]. Previous alignment methods [11,12,13,14,15] used high end INS, such as fiber optic gyro (FOG) strapdown inertial navigation system (SINS), attaining high alignment accuracy. However, the alignment of MEMS IMUs cannot be directly achieved using these methods because MEMS inertial sensors have a relatively large constant bias, which can seriously affect the alignment accuracy. 

Directly separating the earth’s rotation rate from the outputs is difficult because of the constant drift and noise of MEMS gyros, so the alignment of MEMS INS is always based on information from high end INS, which is the transfer alignment [16,17,18], or by using external aids such as magnetometers or satellite receivers to obtain the heading angle in the static condition [19,20,21,22]. The drawback of the latter method is that the magnetometer is prone to disturbances from electrical and metal surroundings, and satellite signals are not available in urban canyons, or indoor or underwater environments. Some studies researched the alignment of MEMS IMU for UAVs or AUVs assisted by global positioning systems (GPS) or the Doppler velocity log (DVL). Then, a non-linear filter method, such as an unscented Kalman filter (UKF) or a particle filter (PF), was applied to estimate the attitude errors. This method requires a large amount of computation, and the heading accuracy is generally only within a few degrees [23,24,25].

Some articles have been published about using MEMS inertial sensors to find north using single-axis rotation, which is the principle upon which the MEMS gyro north finder is based [26,27,28]. Applying RMT to the MEMS gyro north finder provides a new method for the initial alignment of the MEMS IMU. The MEMS gyro north finder can only find north with a static base; when the base is swinging or vibrating, the error is large and the outputs are not available because the MEMS gyro north finder usually has only one single-axis gyro and one or two accelerometers [4]. A single axis gyro cannot track the changes in the attitude angles of the swing base, but the MEMS IMU has three gyros and three accelerometers, so theoretically, the attitude change can be tracked. The MEMS gyro has a relatively large constant drift, but the equivalent drift of the east gyro has the greatest impact on heading accuracy according to the alignment accuracy limit formula [29,30]. The RMT mitigates or even eliminates the equivalent east or north error of the inertial sensors, so aligning the MEMS IMU under swing conditions is possible.

By rotating the IMU using a pre-defined rotation strategy, rather than using external assisting information, the errors of the inertial sensors can be mitigated, eventually improving the IMU performance [5]. For example, the inertial sensor bias can be modulated into a periodic signal by rotating the IMU at a certain angular rate, and the errors caused by the inertial sensor bias can be corrected during a complete rotation cycle [3,4]. Some researchers have studied the performance of rotary MEMS IMU [5,31,32]. Although the introduction of RMT inevitably increases the complexity and cost of the system, MEMS IMU perform better by using the existing rotary platform. A low-cost MEMS IMU provided a good navigation solution by being attached to the wheel of the ground vehicle [33]. For rotary MEMS IMUs, many researchers have conducted simulation tests, theoretical analyses, or verified its superior navigation performance compared with non-rotating IMUs.

To the best of our knowledge, the alignment of rotary MEMS IMU in the swing condition has not been attempted. With the improvement of the accuracy of MEMS inertial sensors and the introduction of RMT, aligning MEMS IMU without external aiding information is feasible. Therefore, the inertial frame-based alignment method using the rotation modulation technique (RMT-IFBA) was used to coarsely align the MEMS IMU on swing base in this paper. Notably, the coarse alignment of the INS, based on a fiber optical gyro (FOG) or other high-precision gyro under swing or moving conditions, could attain high levels of accuracy, but due to the relatively large random noise of the MEMS IMU, fine alignment is required. Alternately, some filtering algorithms could be applied to achieve better accuracy. Frequently used filtering methods include Kalman filter and its improved algorithms. Since the Kalman filter assumes that the system noise is Gaussian white noise, but the actual system noise also includes colored noise, the improved Kalman filter algorithms, such as strong tracking filter (STF), adaptive Kalman filtering (AKF), and robust Kalman filter (RKF), achieve better results for engineering applications [34,35,36,37]. In this paper, the STF, which is robust and adjusts the filtering parameters in real time, was used for fine alignment. 

The purpose of this paper was to study the feasibility of achieving self-alignment of rotary MEMS IMUs in practical applications. The main goal was to achieve a certain alignment accuracy under swing conditions while meeting the requirements for practical applications. To achieve the objective of self-alignment, we propose the use of RMT to reduce the impact of inertial sensor errors on alignment accuracy. The RMT-IFBA method was applied for coarsely aligning under swing conditions, then the STF was used to suppress the random noise of the inertial sensors, which further improved the alignment accuracy of the MEMS IMU. The comparison between the standard Kalman filter and the proposed filtering method verified that the latter had a better effect.

The remainder of this paper is structured as follows: The definition of coordinate frames required in this paper is illustrated in Section 2; the RMT principle and the rotation scheme selected in this paper are described in Section 3; the steps for the RMT-IFBA and the STF method are discussed in Section 4; the simulation test and physical experiment are described in Section 5; and Section 6 draws the conclusions.

## 2. Coordinate Frame Definition

The coordinate frames used in this paper are orthogonal, with the rules of the right-handed coordinate frame being defined. The specific definitions of the different coordinate frames are as follows:

(1) *e* frame: Earth-fixed frame, with its *x*- and *y*-axes fixed on the equatorial plane, with the *z*-axis fixed along the rotational axis of the earth.
(2) *i* frame: Inertial frame, formed by the solidification of the earth coordinate system in inertial space.
(3) *n* frame: Navigation frame. In this work, its *x*-, *y*-, and *z*-axes point to the local east, north, and upward, respectively, which are used for the navigation and attitude representation calculations.
(4) *b* frame: Body frame. Its origin is at the center of the IMU and the *x*-, *y*-, and *z*-axes point to the right, front, and upward, respectively.
(5) *s* frame: IMU frame. Its axes coincide with the sensitive axes of the inertial sensors, and its origin is defined as the origin of the IMU. Additionally, the *s* frame turns in sync with the rotation of the IMU.
(6) *ib*_0_ frame: Body inertial frame, formed by fixing the *b* frame in inertial space at the initial time of the inertial alignment process.

**Figure 1:**
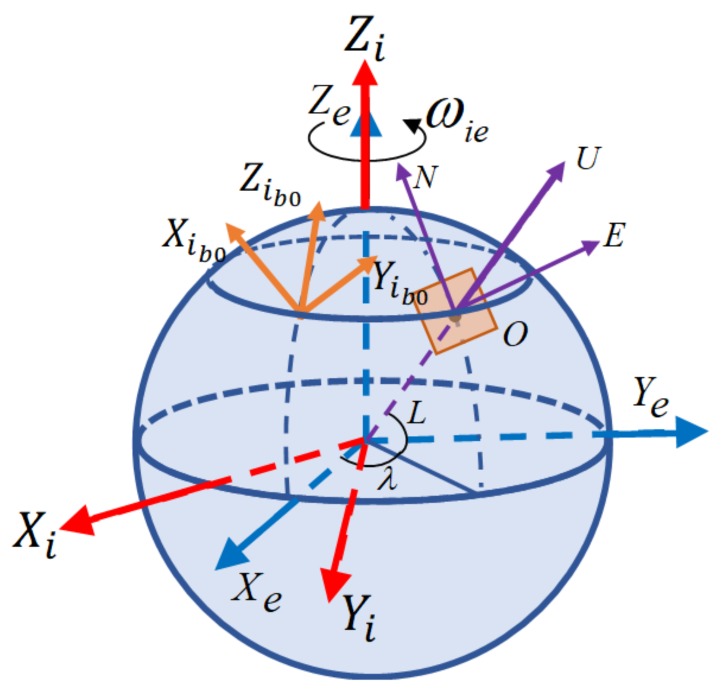
The definition of the coordinate frames.

The different coordinate frames are illustrated in Figure 1.

## 3. Rotation Modulation Technique

**Figure 2:**
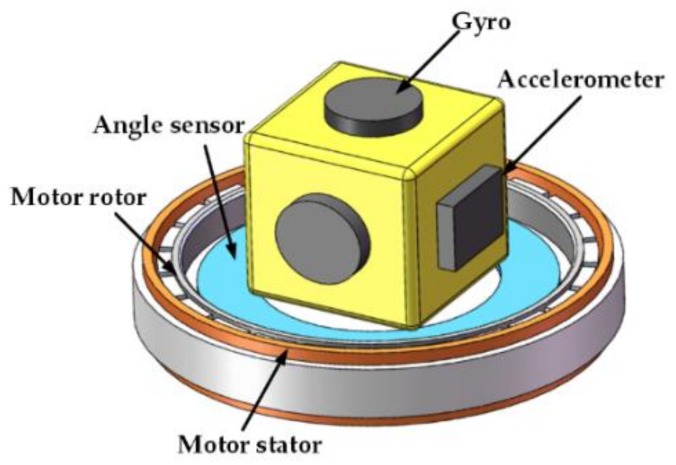
A schematic diagram of a rotary inertial navigation system (INS).

The concept of rotary INS originates from the platform inertial navigation system (PINS) [38]. With the development of inertial technology, the strapdown inertial navigation system (SINS) has become a major trend in INS. A good review of SINS technology was completed by Titterton and Weston [39]. The RMT was applied to SINS and the effect of inertial sensor errors was mitigated by rotating the IMU, and the rotary SINS, based on FOG or laser gyro, has been extensively applied to warships and submarines [40,41,42]. Additionally, the RMT has been studied for the MEMS IMU [31,32,33,43]. Generally, the difference between the rotating IMU and the non-rotating IMU, referred as the conventional IMU, is that the former has a rotating mechanism composed of a motor and an angle sensor for measuring the rotation angle. Its schematic diagram is shown in Figure 2.

### 3.1. Rotation Modulation Technique Principle

To simplify the analysis, the RMT principle was analyzed in the static condition, and we assumed that the body frame was aligned with the navigation frame. RMT analysis requires the introduction of a new coordinate frame, which is the *s* frame defined in Section 2. 

**Figure 3:**
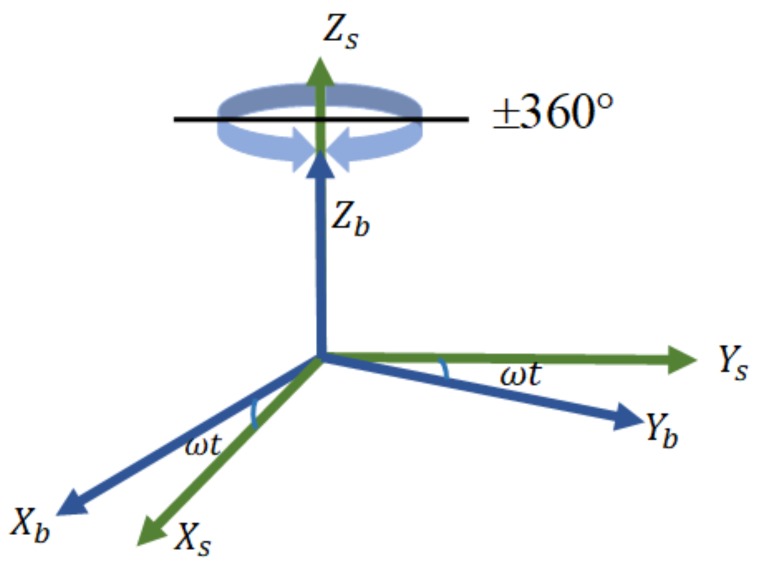
Demonstration of the rotation modulation (RM) technique.

As shown in Figure 3, when the IMU rotates counterclockwise in the positive direction about the *z*-axis at a rotation rate ω, and the *s* frame is initially aligned with the *b* frame, then the transformation matrix from the *s* frame to *b* frame can be expressed as:

(1) $$C_{s}^{b}=\left[\begin{array}{ccc} \cos\omega t & -\sin\omega t & 0\\ \sin\omega t & \cos\omega t & 0\\ 0 & 0 & 1 \end{array}\right]$$

where *t* is the rotation time.

When the IMU is rotating clockwise in the negative direction, the transformation matrix can be described by:

(2) $$C_{s}^{b}=\left[\begin{array}{ccc} \cos\omega t & \sin\omega t & 0\\ -\sin\omega t & \cos\omega t & 0\\ 0 & 0 & 1 \end{array}\right]$$

The transformation matrices of the positive and negative rotation are basically the same, except for the different signs.

As only the signs are different, only the IMU rotating in the positive direction around the *z*-axis is analyzed here. Then, the sensor errors in the *n* frame at time *t* can be expressed as follows:

(3) $$\varepsilon^{n}=C_{b}^{n}C_{s}^{b}\varepsilon^{s}=\left[\begin{array}{ccc} \cos(\omega t) & -\sin(\omega t) & 0\\ \sin(\omega t) & \cos(\omega t) & 0\\ 0 & 0 & 1 \end{array}\right]\left[\begin{array}{c} \varepsilon_{x}^{s}\\ \varepsilon_{y}^{s}\\ \varepsilon_{z}^{s} \end{array}\right]=\left[\begin{array}{c} \varepsilon_{x}^{s}\cos(\omega t)-\varepsilon_{y}^{s}\sin(\omega t)\\ \varepsilon_{x}^{s}\sin(\omega t)+\varepsilon_{y}^{s}\cos(\omega t)\\ \varepsilon_{z}^{s} \end{array}\right]$$

(4) $$\nabla^{n}=C_{b}^{n}C_{s}^{b}\nabla^{s}=\left[\begin{array}{ccc} \cos(\omega t) & -\sin(\omega t) & 0\\ \sin(\omega t) & \cos(\omega t) & 0\\ 0 & 0 & 1 \end{array}\right]\left[\begin{array}{c} \nabla_{x}^{s}\\ \nabla_{y}^{s}\\ \nabla_{z}^{s} \end{array}\right]=\left[\begin{array}{c} \nabla_{x}^{s}\cos(\omega t)-\nabla_{y}^{s}\sin(\omega t)\\ \nabla_{x}^{s}\sin(\omega t)+\nabla_{y}^{s}\cos(\omega t)\\ \nabla_{z}^{s} \end{array}\right]$$

where $\varepsilon^{s}={\left[\begin{array}{ccc} \varepsilon_{x}^{s} & \varepsilon_{y}^{s} & \varepsilon_{z}^{s} \end{array}\right]}^{T}$ and $\nabla^{s}={\left[\begin{array}{ccc} \nabla_{x}^{s} & \nabla_{y}^{s} & \nabla_{z}^{s} \end{array}\right]}^{T}$ are the gyro constant drift and accelerometer bias represented in *s* frame, respectively; and $\varepsilon^{n}$ and $\nabla^{n}$ are the gyro constant drift and accelerometer bias represented in *n* frame, respectively. 

In a complete rotation cycle, the integration of the gyro drift and the accelerometer bias in *n* frame can yield the following formulas:

(5) $$\int_{0}^{T}\varepsilon^{n}dt=\left[\begin{array}{c} 0\\ 0\\ T\varepsilon_{z}^{s} \end{array}\right]$$

(6) $$\int_{0}^{T}\nabla^{n}dt=\left[\begin{array}{c} 0\\ 0\\ T\nabla_{z}^{s} \end{array}\right]$$

where *T* is the time of a complete rotation cycle.

The constant biases of the inertial sensors perpendicular to the axis of rotation are modulated into periodic signals in the form of a sine or cosine function through the rotation of the IMU, so that their integration for an entire rotation cycle is zero. This is the basic RMT principle. The inertial sensor bias is a slowly varying random variable rather than a constant, so integrating the modulated signal is not exactly zero, but is close to zero for a complete rotation cycle. In more detail, the errors of the inertial sensors are composed of constant biases and random noises, and the constant biases can lead to large errors in the calculation of the attitude and other navigation parameters. The RMT considerably reduces the error caused by constant biases, thus improving the calculation accuracy. As inertial sensor error along the rotation axis cannot be modulated, its integration is proportional to the time, or the error still obeys the law of error propagation for non-rotating INSs. Notably, the errors of the inertial sensors themselves do not disappear, but by using RMT, the errors can be mitigated through integration. For inertial navigation parameters, such as attitude angle, the calculation itself is an integral process, so the use of RMT improves INS accuracy.

### 3.2. Rotation Scheme Design

Given the characteristics of the MEMS inertial sensors, designing an appropriate rotation scheme for the MEMS IMU was necessary. The steps for this process are as follows:

(1) Number of rotating axes. Single axis rotation and dual axis rotation are both common rotation processes. Single axis rotation is inexpensive with a simple structure and high reliability, but it cannot modulate all constant errors. Dual axis rotation can modulate all constant errors, but the complexity and cost are greater. Considering the dual axis rotation scheme reduces the advantages of MEMS IMU, such as its low cost and small size, we decided to use the single axis rotation scheme.
(2) Selection of the rotation axis. The selection of the rotation axis for MEMS IMU must consider that the error along the rotation axis cannot be modulated. Therefore, considering which axis rotation can minimize the effect of unmodulated errors on attitude is important. In the literature [5], gyro errors in the horizontal plane cause a larger attitude error, so the rotation scheme around the *z*-axis is better than the rotation around the *x*- or *y*-axis. Therefore, we chose the rotation scheme around the *z*-axis.
(3) Rotation direction. Two single axis rotation schemes, namely unidirectional rotation and reciprocation rotation, were discussed. The scale factors for gyros and accelerometers are usually considered to be constant in a relatively stable environment for a short time [5,44]. However, due to the existence of residual errors in the scale factor, the angular rate of unidirectional rotation coupled with the scale factor error creates a larger error. In contrast, the reciprocation rotation can offset the error caused by the scale factor [32]. As shown in Figure 3, a complete reciprocation rotation cycle includes 360 degrees of rotation about the *z*-axis in the counterclockwise (positive) direction, and then a rotation cycle in the clockwise (negative) direction. This method has been used in previous work [32,45], and was also used in this paper.
(4) Rotation continuity. The reciprocation rotation scheme can be used for continuous rotation or an alternating rotating-stopping scheme. During continuous rotation, the constant error is modulated into a cosine signal. The alternate rotating-stopping scheme rotates a certain angle and stops at the current position before continuing to rotate to the next position. For high-end INS, the short-term stop time will not significantly impact the navigation error, but for the MEMS IMU, the navigation error be significantly increases when rotation stops [46]. Therefore, we used a continuous rotation scheme in this paper.
(5) Angular rate of rotation. The RMT effect is related to the angular rate of rotation. Theoretically, the faster the rate of rotation, the more obvious the effect of error suppression [47,48]. However, for practical engineering applications, a too fast angular rate of rotation cause control difficulties, high frequency noise, and additional error caused by the motor. For high-end INS, a smaller rotational angular rate allows more precise control of the motor. Regardless, a high rotation rate can significantly inhibit the effect of the errors of MEMS inertial sensors. Therefore, the effect of error suppression should be considered, along with avoiding the direction transformation errors caused by the excessive rotation rate. Accordingly, a comprehensive consideration of the above factors and for convenience of calculation, the simulation and experimental rotational angular rate was set to 20°/s.

## 4. Rotating MEMS IMU Alignment Principle

**Figure 4:**
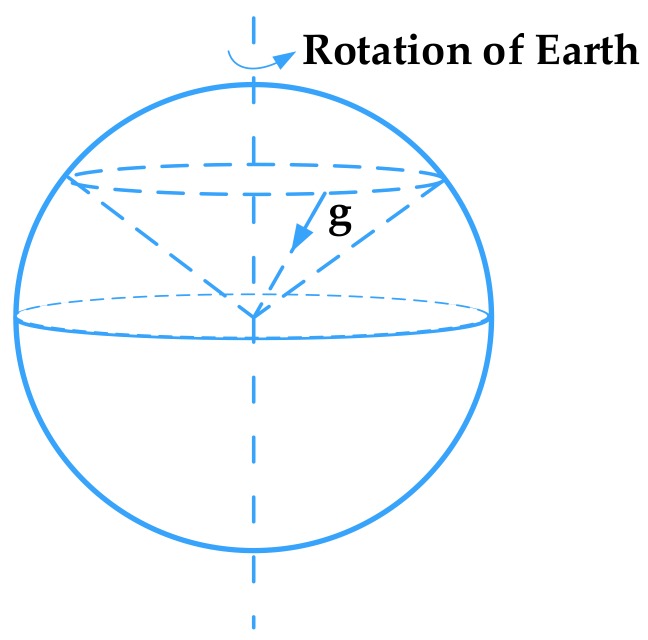
Conical movements of the gravity vector in inertial space.

Due to the earth’s rotation, the pure gravitation vector in inertial space is capable of forming a cone, and geomorphic north can be determined by observing the change in direction of the gravity in inertial space, as shown in Figure 4. By mapping the gyro and accelerometer measurements into inertial space, the pure gravitational vector and the rotation rate of the earth used for the alignment can be obtained after the periodic disturbance is smoothed [49,50]. As explained in Section 1, the alignment of the rotating MEMS IMU under swing conditions includes both coarse and fine alignment. 

### 4.1. Coarse Alignment

To coarsely align the MEMS IMU, we introduced the RMT-IFBA method. At the initial moment of the alignment, the *s* frame is aligned with the *ib*_0_ frame. When the alignment begins, the *ib*_0_ frame is solidified in inertial space and the *s* frame starts to rotate periodically, and the transformation matrix from the *s* frame to the *n* frame can be expressed by:

(7) $$C_{s}^{n}=C_{i}^{n}C_{i_{b0}}^{i}C_{s}^{i_{b0}}$$

(1) According to the latitude *L* of the location and the time interval Δt of the alignment, the transformation matrix from the *i* frame to the *n* frame can be determined.

(8) $$C_{i}^{n}=C_{e}^{n}C_{i}^{e}=\left[\begin{array}{ccc} -\sin\omega_{ie}\Delta t & \sin\omega_{ie}\Delta t & 0\\ -\sin L\cos\omega_{ie}\Delta t & -\sin L\sin\omega_{ie}\Delta t & \cos L\\ \cos L\cos\omega_{ie}\Delta t & \cos L\sin\omega_{ie}\Delta t & \sin L \end{array}\right]$$

where $\omega_{ie}$ is the earth’s rotation rate.

(2) The transformation matrix from the *ib*_0_ frame to the *s* frame can be expressed as:

(9) $$C_{s}^{i_{b0}}=C_{b}^{i_{b0}}C_{s}^{b}$$

As shown in Equation (10), in the swing condition, the output of the gyros includes the earth’s rotation rate, the angular rate caused by the swing base, the rotation rate of the IMU, and the gyro drift. Due to the introduction of the RMT, the impact of the gyro drift is drastically reduced.

(10) $$\omega_{i_{b0}s}^{s}=\omega_{ib_{0}s}^{s}+\delta \omega^{s}+\omega_{bs}^{s}+\varepsilon^{s}$$

The quaternion method is used to update the direction cosine matrix (DCM) differential equation shown in Equation (11):

(11) $${\dot{C}}_{s}^{i_{b0}}=C_{s}^{i_{b0}}[\omega_{i_{b0}s}^{s}\times ].$$

(3) Determine the transformation matrix from the *ib*_0_ frame to the *i* frame. Due to the rotation of the earth, the gravity in inertial space varies with time, which can be expressed as:

(12) $$g^{i}=\left[\begin{array}{c} -g\cos L\cos\omega_{ie}\Delta t_{k}\\ -g\cos L\sin\omega_{ie}\Delta t_{k}\\ -g\sin L \end{array}\right]$$

where $\Delta t_{k}=t_{k}-t_{0}$ is the time interval.

The velocity value corresponding to the gravity at time $t_{k}$ can be obtained by integrating the gravity in the *i* frame, which is shown as follows:

(13) $$V^{i}(t_{k})=\int_{t_{0}}^{t_{k}}g^{i}dt=\left[\begin{array}{c} \frac{g\cos L\sin\omega_{ie}\Delta t_{k}}{\omega_{ie}}\\ \frac{g\cos L[1-\cos\omega_{ie}\Delta t_{k}]}{\omega_{ie}}\\ g\sin L\Delta t_{k} \end{array}\right].$$

As represented in Equation (14), the output of the accelerometers includes the gravity in *s* frame, the disturbance acceleration $\delta a_{s}$ caused by the swing base, and the accelerometer bias.

(14) $$f^{s}=-g^{s}+\delta a^{s}+\nabla^{s}$$

The accelerometer bias is converted into a periodic signal when the IMU rotates about the *z*-axis, so the velocity value corresponding to the measured acceleration in the *ib*_0_ frame can be expressed as:

(15) $$\begin{array}{cc} V^{i_{b0}} & =\int_{t_{0}}^{t_{k}}f^{i_{b0}}dt\\ & =\int_{t_{0}}^{t_{k}}C_{s}^{i_{b0}}f^{s}dt\\ & =\int_{t_{0}}^{t_{k}}C_{s}^{i_{b0}}(-g^{s}+\delta a^{s})dt\\ & =-C_{i}^{i_{b0}}\int_{t_{0}}^{t_{k}}g^{i}dt+\int_{t_{0}}^{t_{k}}C_{s}^{i_{b0}}\delta a^{s}dt \end{array}$$

Define $V^{i}=-\int_{t_{0}}^{t_{k}}g^{i}dt$ and $\Delta V^{i_{b0}}=\int_{t_{0}}^{t_{k}}C_{s}^{i_{b0}}\delta a^{s}dt$. Because the disturbance acceleration $\delta a_{s}$ is an approximate periodic signal, the velocity error corresponding to its integration is close to zero. Then the transformation matrix $C_{i_{b0}}^{i}$ can be acquired using Equation (16).

(16) $$V^{i}=C_{i_{b0}}^{i}V^{i_{b0}}$$

The velocity values, $V(t_{k1})$ and $V(t_{k2})$, at time $t_{k1}$ and $t_{k2}$ are used to construct two auxiliary vectors, $V(t_{k1})\times V(t_{k2})$ and $V(t_{k1})\times V(t_{k2})\times V(t_{k1})$. Then the solution for $C_{i_{b0}}^{i}$ can be described as:

(17) $$C_{i_{b0}}^{i}={\left[\begin{array}{c} {\left[V^{i}(t_{k1})\right]}^{T}\\ {\left[V^{i}(t_{k1})\times V^{i}(t_{k2})\right]}^{T}\\ {\left[V^{i}(t_{k1})\times V^{i}(t_{k2})\times V^{i}(t_{k1})\right]}^{T} \end{array}\right]}^{-1}\left[\begin{array}{c} {\left[V^{i_{b0}}(t_{k1})\right]}^{T}\\ {\left[V^{i_{b0}}(t_{k1})\times V^{i_{b0}}(t_{k2})\right]}^{T}\\ {\left[V^{i_{b0}}(t_{k1})\times V^{i_{b0}}(t_{k2})\times V^{i_{b0}}(t_{k1})\right]}^{T} \end{array}\right]$$

(4) The coarse alignment matrix result $C_{s}^{n}$ can be obtained by substituting $C_{i}^{n}$, $C_{s}^{ib_{0}}$, and $C_{ib_{0}}^{i}$ from Equations (8), (11), and (17), respectively, into Equation (18):

(18) $$C_{s}^{n}=C_{i}^{n}C_{i_{b0}}^{i}C_{s}^{i_{b0}}$$

**Figure 5:**
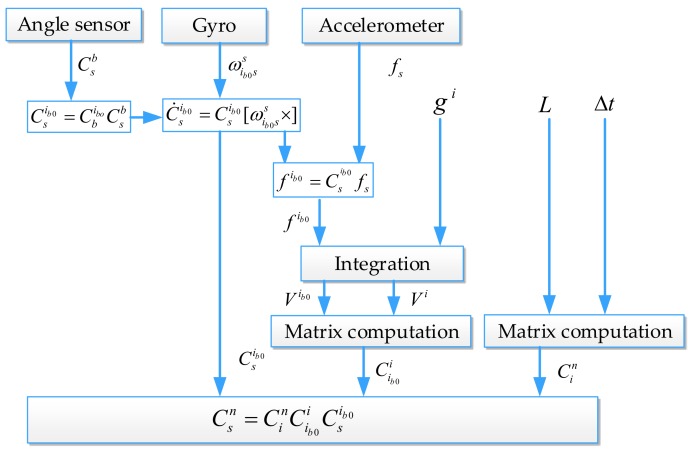
The principle of the inertial frame-based alignment using the rotation modulation technique (RMT-IFBA) scheme.

Based on the above analysis, the principle of the RMT-IFBA scheme is illustrated in Figure 5.

### 4.2. Fine Alignment

The actual measured data of MEMS IMU have random noise; therefore, fine alignment was conducted to further improve the alignment accuracy. The Kalman filter and its improved algorithms are widely used in engineering applications because of their efficient noise reduction. The Kalman filter is a linear system-based optimal estimation filter that assumes the noise is Gaussian white noise. However, the real system model often has some uncertainties and different degrees of nonlinearity, and the noise of inertial sensors is not entirely white noise. Therefore, the standard Kalman filter is not always an optimal estimation method for practical systems, and improved Kalman filter algorithms are often used in engineering applications [51]. This paper adopted the STF algorithm previously proposed [34] to reduce the effect of random noise. The STF algorithm is an improved algorithm for Kalman filter, and it has been widely used for fault detection, adaptive control, and in other fields [52,53].

The key step in designing a Kalman filter or STF is the establishment of the state space model and the measurement model. Since the IMU is attached to the swing base, its velocity and position should be zero, so the velocity error can be considered an observation. Then the state space model and the measurement model can be described as follows, respectively:

(19) $$\dot{x}(t)=F(t)x(t)+G(t)w(t)$$

(20) z(t)=H(t)x(t)+v(t)

where $F(t)=\left[\begin{array}{c} \begin{array}{c} \begin{array}{cccc} -\omega_{ie}^{n}\times & 0_{3\times 3} & -C_{s}^{n} & 0_{3\times 3} \end{array}\\ \begin{array}{cccc} -g^{n}\times & 0_{3\times 3} & 0_{3\times 3} & -C_{s}^{n} \end{array} \end{array}\\ 0_{6\times 12} \end{array}\right]$, $\omega_{ie}^{n}\times$ is the antisymmetric matrix of the earth’s rotation rate in the *n* frame, $g^{n}\times$ is the antisymmetric matrix of local gravity in the *n* frame, $C_{s}^{n}$ is the transformation matrix from the *s* frame to the *n* , $G=\left[\begin{array}{c} \begin{array}{cc} -C_{s}^{n} & 0_{3\times 3} \end{array}\\ \begin{array}{cc} 0_{3\times 3} & C_{s}^{n} \end{array}\\ 0_{6\times 6} \end{array}\right]$, w(t) is the intrinsic noise of the inertial sensors, and v(t) is the measurement noise.

The state vector is $x={\left[\begin{array}{ccc} \begin{array}{cccc} \begin{array}{cccc} \begin{array}{cccc} \phi_{E} & \phi_{N} & \phi_{U} & \delta V_{E} \end{array} & \delta V_{N} & \delta V_{U} & \varepsilon_{x} \end{array} & \varepsilon_{y} & \varepsilon_{z} & \nabla_{x} \end{array} & \nabla_{y} & \nabla_{z} \end{array}\right]}^{T}$, where $\phi_{i}$ (*I = E,N,U*) is the misalignment angle vector, $\delta V_{i}$ (*i = E,N,U*) is the velocity-error vector, $\varepsilon_{i}$ *(i = x, y, z*) is the gyro drift vector, and $\nabla_{i}$ *(i = x, y, z*) is the acceleration bias vector. The measurement matrix is $H=\left[\begin{array}{cccc} 0_{3\times 3} & I_{3\times 3} & 0_{3\times 3} & 0_{3\times 3} \end{array}\right]$.

In the Kalman filter, the system noise and the measurement noise are considered uncertainties, and the recursive formulas of the Kalman filter are as follows:

(21) $$X_{k+1,k}=FX_{k,k}$$

(22) $$P_{k+1,k}=FP_{k,k}F^{T}+GQ_{k}G^{T}$$

(23) $$K_{k+1}=P_{k+1,k}H^{T}{\left(HP_{k+1,k}H^{T}+R_{k+1}\right)}^{-1}$$

(24) $$X_{k+1,k+1}=X_{k+1,k}+K_{k+1}(Z_{k+1}-HX_{k+1,k})$$

(25) $$P_{k+1}=(I-K_{k+1}H)P_{k+1,k}$$

where $X_{k,k}$ is the state estimated at time *k,* $X_{k+1,k}$ is the one-step prediction of the state, *F is* the one-step state transformation matrix, *H* is the measurement matrix, $P_{k,k}$ is the error covariance matrix at time *k*, $P_{k+1,k}$ is the predicted error covariance matrix, $K_{k+1}$ is the gain vector at time *k* + 1, *G* is the system noise driven matrix, $Q_{k}$ is the covariance matrix of the system noise, and $R_{k+1}$ is the covariance matrix of the measurement noise at time *k* + 1.

For the STF, the recursive formulas are basically the same as for the Kalman filter except for the one-step prediction of the mean square error formula. This STF formula is:

(26) $$P_{k+1/k}=F_{k+1/k}\sqrt{D_{k+1}}P_{k}\sqrt{D_{k+1}}F_{k+1/k}^{T}+G_{k}Q_{k}G_{k}^{T}$$

where $D_{k+1}$ is a diagonal matrix of fading factors at time *k* + 1. 

Obviously, the STF formula degrades to a standard Kalman filter when all the fading factors are one. The solution method for multiple fading factors has been previously described [34,52]. The mean square error matrix, the *P* matrix, is dynamically adjusted, so it has better robustness and state tracking ability [53,54].

**Figure 6:**
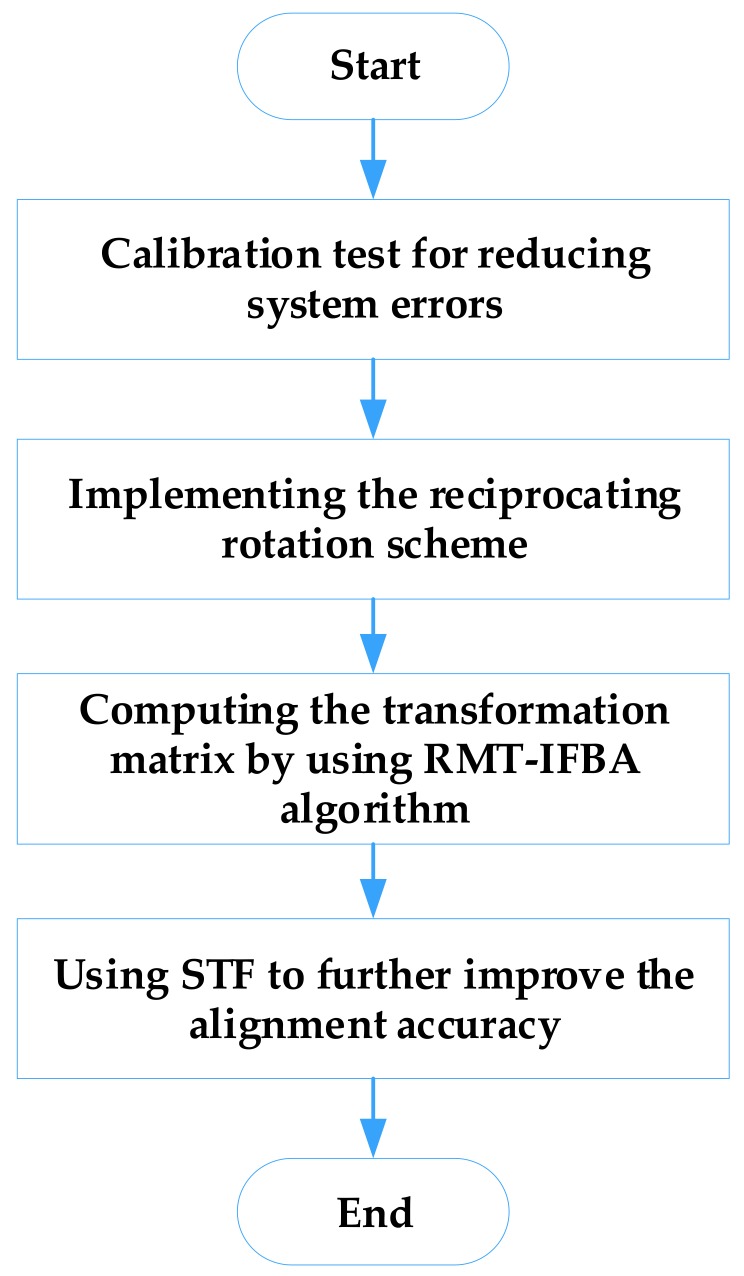
Flow chart of the main procedures of the proposed method.

Based on the above description, the main procedures of the proposed method for the alignment of MEMS IMU in the swing condition are depicted in Figure 6.

## 5. Simulation and Experimental Analysis

In this section, the simulation and physical experiments are designed to evaluate the method used to align a rotary MEMS IMU under the swing base condition. The simulation test was first implemented to theoretically demonstrate the feasibility of the method. As the simulation condition was relatively ideal and uncomplicated, only the coarse alignment process was conducted. Both coarse and fine alignment were completed for the turntable experiment, and the result of the proposed method was compared with that of a high-end INS to validate the performance of the proposed method.

### 5.1. Simulation Test

In this subsection, the alignment of the rotating MEMS IMU with a swing base was studied to theoretically verify that MEMS IMU could achieve certain alignment accuracy using the RMT-IFBA algorithm.

The swing rule was Asin(2πt/T+φ)+θ, and *A* and *T* were the amplitude and cycle of the swing base, respectively, whereas φ was uniformly distributed around 0, 2π was a random initial phase, and θ represented the swing center. The swing parameters for the simulation test are shown in Table 1.

The alignment time of this test was 300 s, and the geographic latitude and longitude of the MEMS IMU were set as *L* = 40° and λ = 120°. The inertial sensor errors are set in Table 2.

The data sampling period was 0.01 s, the reciprocating rotation rate was 20°/s, and $t_{k1}$ and $t_{k2}$ were 50 s and 250 s, respectively. The alignment was performed 50 times. In the simulation test, we simulated the motion of the ship under mooring conditions according to the swing parameters in Table 1 and obtained the IMU data. The errors shown in Table 2 were introduced into the data. According to Equations (8)–(18), the alignment was solved using the RMT-IFBA method, and the results of the calculation were compared with the actual attitude of the ship to obtain the alignment error. To compare the effects of the rotation modulation technique (RMT) around different axes, we simulated the alignment of the IMU around the rotation of the *x*-, *y*-, and *z*-axes. Notably, the transformation matrix was not the same for the rotation around different axes. In this paper, the transformation matrix around the *z*-axis is presented in Equations (1) and (2). The transformation matrix corresponding to the rotation about the *x*- and *y*-axes was provided by Shuang Du [5]. 

**Figure 7:**
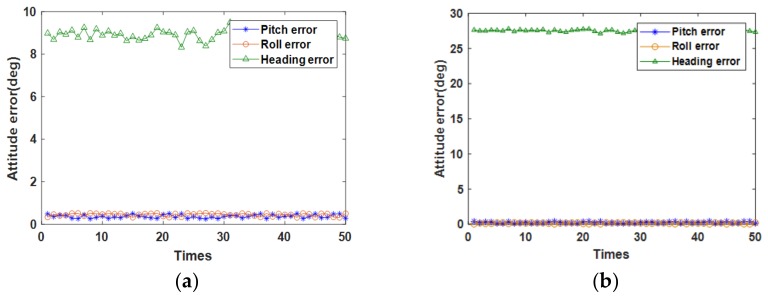

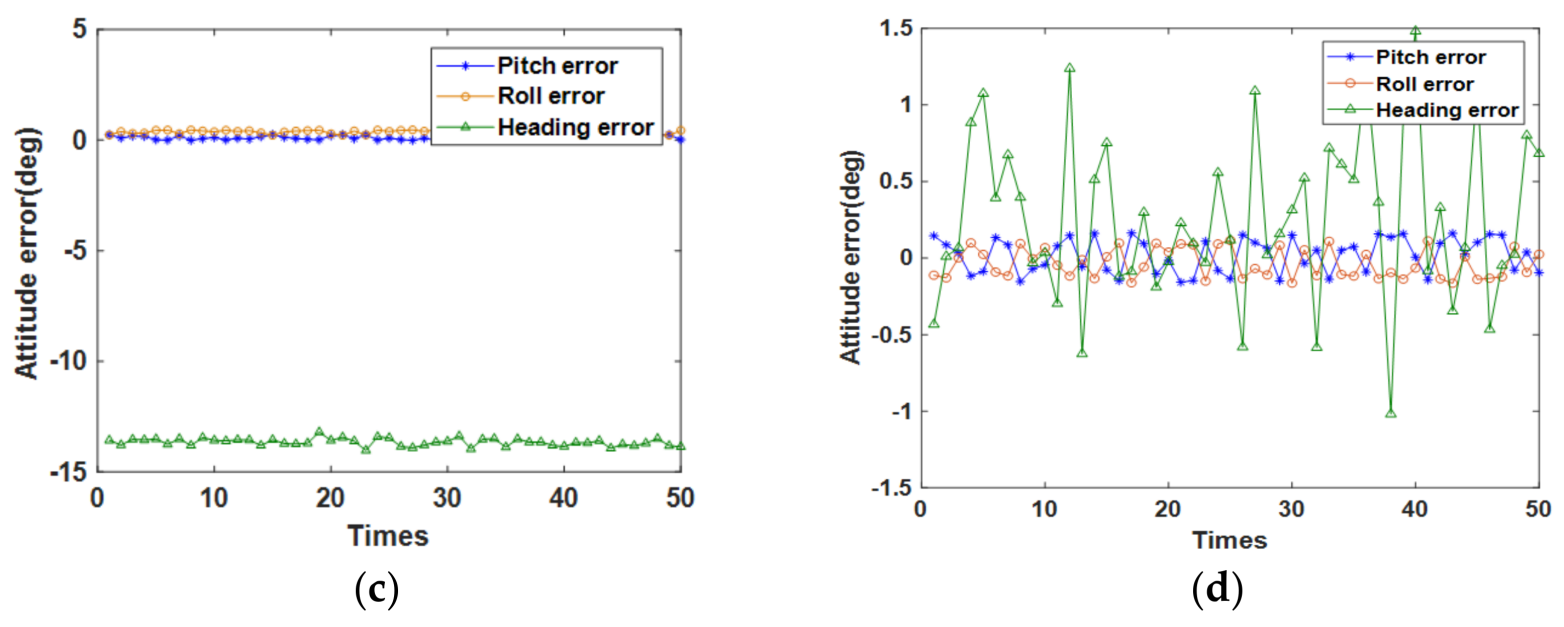
The alignment attitude errors for (**a**) the non-rotating inertial measurement unit (IMU), (**b**) the MEMS IMU rotating around the *x*-axis, (**c**) the MEMS IMU rotating around the *y*-axis, and (**d**) the MEMS IMU rotating around the *z*-axis.

As shown in Figure 7, considerable error occurred in the heading angle for the non-rotating MEMS IMU. If the MEMS IMU rotated around the *x*- or *y*-axis, the alignment result would also have a large error. When the *z*-axis was the axis of rotation, the RMT significantly improved the alignment accuracy. The comparison determined it was theoretically feasible to align the MEMS IMU under swing conditions by applying RMT rotating around *z*-axis. Explaining the simulation results here is required. Since the calculation of the heading angle is equivalent to north-seeking, and the measurement of the northward information of the earth is easily affected by the bias of the equivalent horizontal gyro, but the rotation of MEMS IMU around the *z*-axis greatly reduces the influence of the constant drift of the horizontal gyro, self-aligning the MEMS IMU rotating around *z*-axis was possible. Additionally, the MEMS gyro north seeker always uses a horizontal gyro and rotates around the *z*-axis to find north. The qualitative analysis mentioned above helps explain the simulation results. In this simulation test, the mean value and standard deviation (SD) of the alignment errors of the rotary MEMS IMU rotating around *z*-axis are listed in Table 3.

Given the simulation test results, it appeared theoretically possible to achieve certain alignment accuracy for the MEMS IMU on a swing base if using RMT. However, the theory had to be experimentally verified to prove the efficacy of the algorithm. Therefore, the next step was to conduct a turntable experiment to test the practicality and reliability of the proposed method.

### 5.2. Physical Experiment Setup

**Figure 8:**
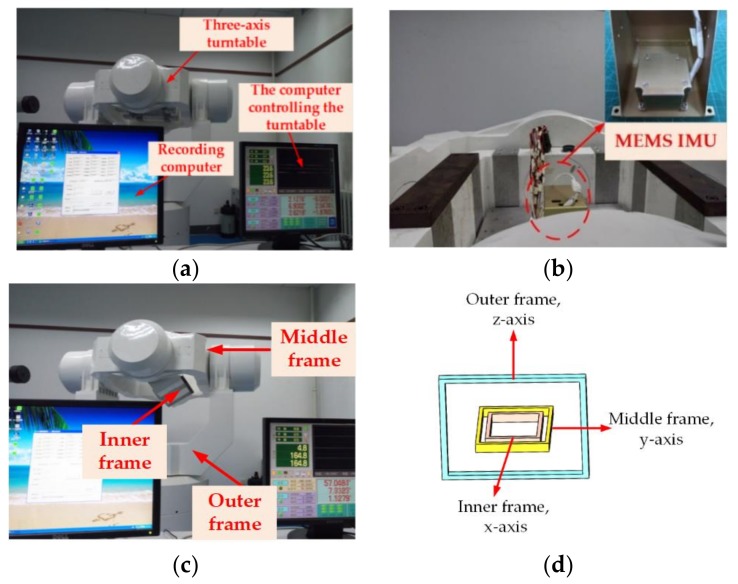
(**a**) The turntable experiment setup. (**b**) The MEMS IMU used in the experiment. (**c**) Description of the turntable frame. (**d**) Schematic diagram of the turntable frame.

The experimental setup is illustrated in Figure 8a. As shown in Figure 8b, the MEMS IMU produced by our laboratory was used to verify the performance of the proposed method for practical applications. The MEMS IMU included three MEMS gyros and three MEMS accelerometers, which were all designed by our laboratory. The specifications of this IMU are listed in Table 4. The description and schematic diagram of the turntable frame are shown in Figure 8c,d, respectively.

The MEMS IMU was fixed on the three-axis turntable, which was designed by the AVIC (Aviation Industry Corporation of China) Beijing Precision Engineering Institute (Beijing, China) to perform the alignment experiment. During the test, the turntable and MEMS IMU data were collected using the serial communication port, and the data sampling rate was 100 Hz. Before the experiment, a calibration test was performed to correct the coupling scale factors of the inertial sensors, installation errors, and g-sensitivity error to avoid the above-mentioned errors.

### 5.3. Allan Variance Method

**Figure 9:**
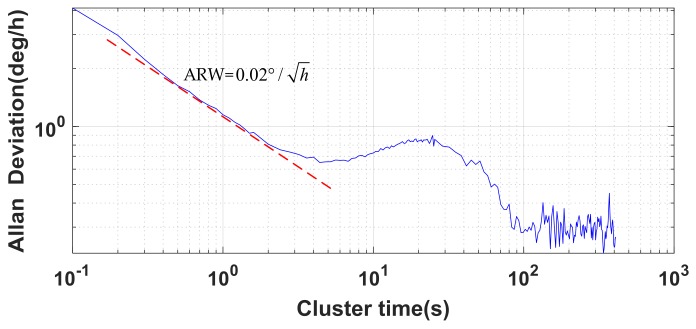
Allan graphic of the gyro data in the *x*-axis.

Allan variance analysis was applied to study the random variations of the gyro, as shown in Figure 9, which shows the Allan variance of the MEMS gyro data in the *x*-axis.

As the cluster time increased, the Allan variance gradually decreased because the random noise was smoothed. The significant reduction after 20 s may be related to the low frequency noise of the gyro from 0.01 to 0.1 Hz, and the specific source may be the noise introduced by the gyro’s circuit-tuning loop. With the increase in cluster time, the influence of noise decreased. At about 120 s, the variance of the random error was the lowest. However, as time continued to increase, the variance in the random errors began to fluctuate, due to the effect of rate random walk and rate ramp [5,55]. Similar results were obtained for the MEMS gyro data in the *y*- and *z*-axes. The angle random walk of the gyro was about 0.02${}^{\circ}/\sqrt{h}$, calculated using the Allan variance formula. The bias stability of the gyro was about 3°/h over 10 s of the smoothing algorithm.

### 5.4. Turntable-Based Alignment Experiment

To achieve a swing state while the three-axis turntable was in a rotation state, we set the parameters of the turntable control program so that the inner frame and the middle frame would swing in sine mode and the outer frame would have a reciprocating rotation. This scheme was called Scheme 1. At the beginning of each experiment, three frames of the turntable initially turned to a static position for about 50 s. The gyro had a larger constant drift when the IMU was on, and this constant value was deduced from the gyro data. The earth rotation rate was subtracted at the same time, so we assumed the gyro data had a constant drift after deducting the constant value corresponding to the turn-on of the gyro. The turntable then entered swing mode with single axis rotation for a duration of about 450 s. The final location of the MEMS IMU was the same as the initial position. The parameters of the swing mode and rotation mode in Scheme 1 are shown in Table 5.

To verify the repeatability of the experiment, a total of 10 experiments were completed, with each experiment lasting about 500 s. For each experiment, $t_{k1}$ was set at the 50th second, while $t_{k2}$ was set at the 300th second when the turntable entered into rotation mode. The pitch, roll, and heading angle of the turntable at the final position were recorded as a reference to compare the results of the proposed alignment scheme.

**Figure 10:**
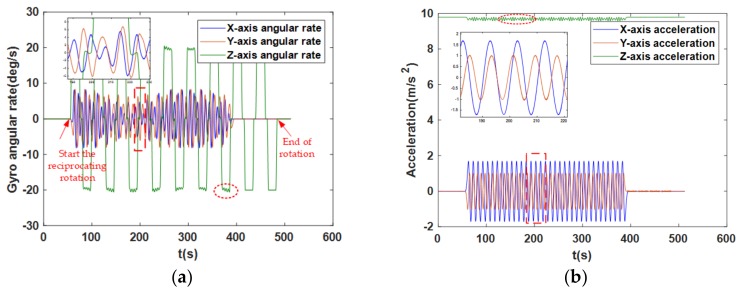
(**a**) The angular rate of the *x*-, *y*-, and *z*-axis in Scheme 1. (**b**) The acceleration of the *x*-, *y*-, and *z*-axis in Scheme 1.

The change in the angular rate and the acceleration of the MEMS IMU in Scheme 1 are displayed in Figure 10. The graphic in the red rectangle dotted box is partially enlarged to provide additional detail. The red ellipse dotted box appeared in the meander curve due to the change in attitude under the swing condition. Specifically, due to the swing state of the *x*- and *y*-axis, the plane formed by the *x*- and *y*-axes was not always a horizontal plane but swung correspondingly. Therefore, the angular rate of the *z*-axis was coupled with some angular rate components caused by the swing, and the acceleration also fluctuated near the local gravitational acceleration because of the swing state. This is consistent with the changing law of angular rate and acceleration under the swing condition.

In addition, we conducted another experimental scheme for comparison under the same experimental conditions. This scheme was called Scheme 2. The swing base was simulated using the outer and middle frame, and the rotation of the IMU was performed at the inner frame. The parameters of the three frames in Scheme 2 are shown in Table 6.

**Figure 11:**
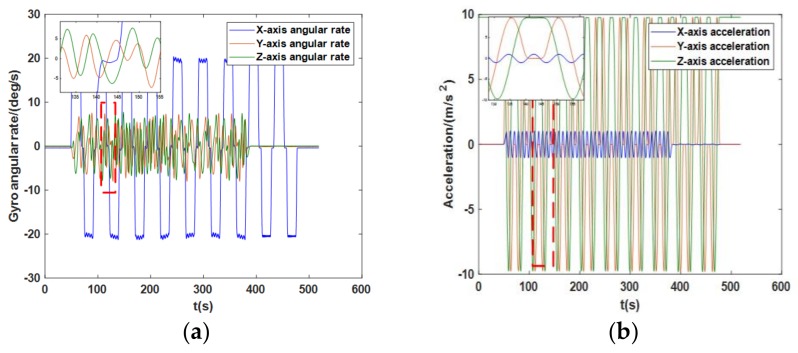
(**a**) The angular rate of the *x*-, *y*-, and *z*-axis in Scheme 2. (**b**) The acceleration of the *x*-, *y*-, and *z*-axis in Scheme 2.

The change in the angular rate and the acceleration of the MEMS IMU in Scheme 2 is shown in Figure 11. Due to the reciprocating rotation around the *x*-axis, the acceleration of the *y*- and *z*-axis periodically changed between ±*g*, and due to the periodic swing motion, the *x*-axis had a periodically varying acceleration until no swing motion occurred.

The initial attitude angles for the three-axis turntable were all set to 0°, and the final location was the same as the initial position in the alignment experiments. However, the turntable in the laboratory was only calibrated in the horizontal plane, but the heading was not calibrated. Therefore, the heading angle displayed on the control computer was not the exact heading angle. As such, a high-end INS, Epsilon20, developed by Safran Electronics and Defense Company (Boulogne-Billancourt, France), was used to obtain the real attitude at the initial position. The INS is based on hemispherical resonator gyro (HRG) technology and provides position and angle information in case of GPS failure. The heading angle obtained by Epsilon20 was used as a reference. 

**Figure 12:**
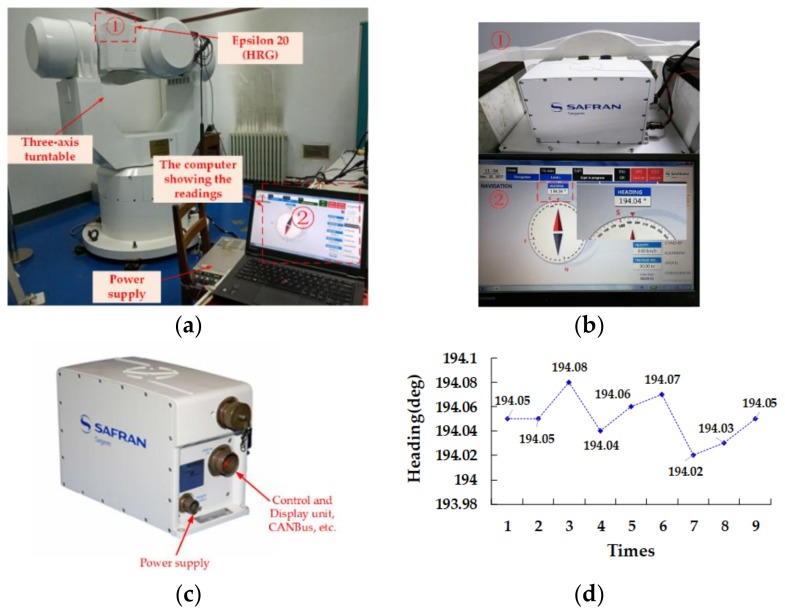
(**a**) The experimental setup with Epsilon 20. (**b**) Partially enlarged detail of (**a**). (**c**) The high-end INS, Epsilon 20, based on hemispherical resonator gyro (HRG) technology. (**d**) Nine sets of heading data collected with Epsilon 20.

As shown in Figure 12, nine groups of repeated experiments were performed at the same position with Epsilon20, and the mean value was about 194°, whereas the SD was 0.04° of the heading angle in the clockwise direction. Due to the positive direction being defined in our algorithm in the counterclockwise direction, whereas Epsilon20 is in the clockwise direction, the heading angle computed in this paper should have been approximately equal to 166°. Based on the above analysis, the calculated pitch angle and roll angle should have been 0°, and the heading angle should have been about 166°. If so, the effectiveness and reliability of the algorithm would be confirmed.

### 5.5. Result Analysis

**Figure 13:**
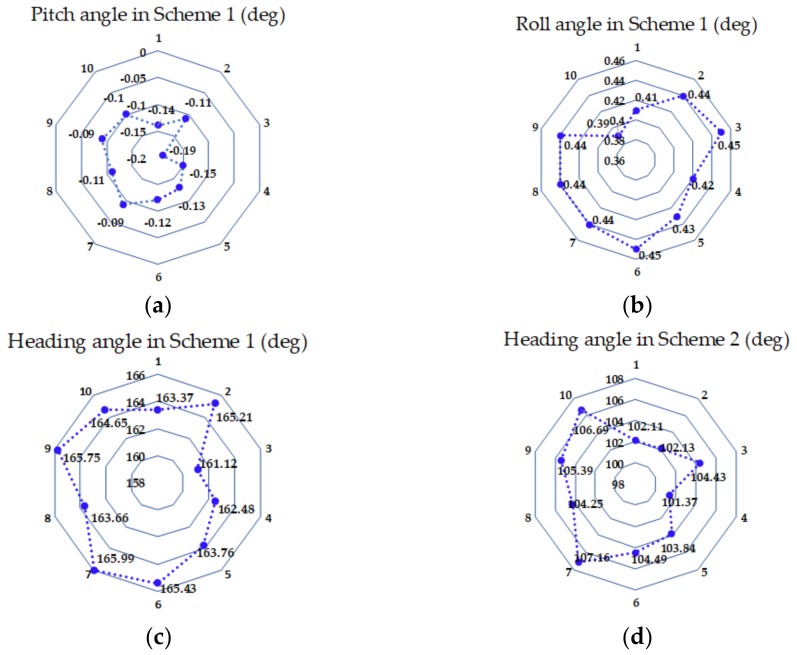
(**a**) The pitch angle of the coarse alignment using the RMT-IFBA method in Scheme 1, (**b**) the roll angles of the coarse alignment in Scheme 1, (**c**) the heading angles of the coarse alignment in Scheme 1, and (**d**) the heading angles of the coarse alignment in Scheme 2.

To illustrate the MEMS IMU data processing process and algorithm analysis in detail, a set of data were specifically described as an example. Figure 10 shows the change in the data of the MEMS IMU in Scheme 1. The data processing period started with the rotation of the *z*-axis to the end of rotation, as shown in Figure 10. The detailed coarse alignment process was discussed in Section 4.1. The results of the coarse alignment in Scheme 1 for 10 groups of data obtained from the turntable experiment are shown in Figure 13a–c. The heading angle calculated in Scheme 2 is displayed in Figure 13d, and the specific values and distribution are displayed in the radar chart. The error of the heading angle obtained in Scheme 2 was very large, which was the same as the conclusion drawn from the simulation test. Therefore, the results of the simulation and turntable experiments confirmed that the alignment accuracy could be greatly improved by rotating the MEMS IMU around the *z*-axis. Based on the above results, the following content mainly discusses Scheme 1. The mean and SD of the attitude angles in Scheme 1 are listed in Table 7.

**Figure 14:**
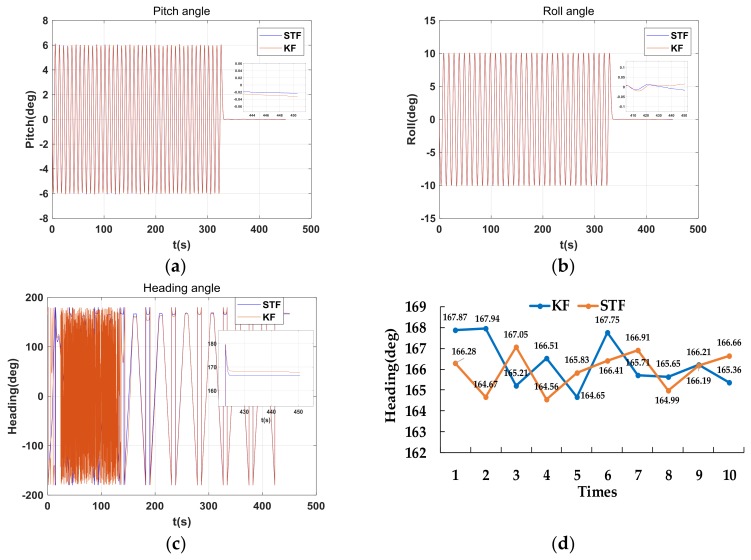
(**a**) The change in pitch angle obtained using Kalman filter (KF) and strong tracking filter (STF), (**b**) the change in roll angle using KF and STF, (**c**) the change in heading angle using KF and STF, and (**d**) 10 sets of heading angles obtained using KF and STF.

From the Table 7, the difference between the attitude angles of the alignment for the rotary MEMS IMU and the real attitude angles can be approximated as small angles, which created a good foundation for fine alignment. The coarse alignment result was the initial value for fine alignment. Two kinds of filtering algorithms were used in this paper: the standard Kalman filter and the STF described in Section 4.2. The effect of the two filtering methods is shown in Figure 14.

The accuracy of the two filtering methods for tracking the changes in pitch and roll angles was very close, but the tracking accuracy of the heading angle was slightly different. The mean and SD of the three attitude angles for Kalman filter (KF) and STF are shown in Table 8.

From the above analysis, the errors of the attitude angles obtained through coarse alignment were small, and we demonstrated that the RMT could reduce the influence of the constant bias of the inertial sensors. The small error angles were beneficial for further improving the accuracy of the alignment by using the filtering methods. Two filtering algorithms used in this paper; both achieved higher accuracy, but the STF had a better effect. This revealed that the MEMS IMU was successfully aligned by using the proposed method and could be applied to practical systems.

### 5.6. Irregularity of Platform Rotation Discussion

Since RMT is the key to realizing self-alignment of the MEMS IMU, discussing the influence of the irregularity of the rotating platform on the effect of RMT is necessary. The motor’s speed fluctuation error and non-orthogonal angle between the rotation axis and the rotation plane are discussed in this subsection.

During the rotation process, the motor’s speed fluctuation error can cause gyro measurement errors. For the MEMS IMU rotating around the *z*-axis, we denoted the rotation rate as ω, and the rotation speed fluctuation was δω. According to Equation (3), gyro errors on the *x*- and *y*-axis in the navigation frame are:

(27) $$\varepsilon_{x}^{n}=\varepsilon_{x}^{s}\cos(\omega +\delta \omega )t-\varepsilon_{y}^{s}\sin(\omega +\delta \omega )t$$

(28) $$\varepsilon_{y}^{n}=\varepsilon_{x}^{s}\sin(\omega +\delta \omega )t+\varepsilon_{y}^{s}\cos(\omega +\delta \omega )t.$$

The speed fluctuation can be considered as noise contained in the rotation rate, so the modulated signal is not necessarily integrated to zero in a rotation period, which reduces the effect of RMT. Therefore, the measures used to reduce the effect of gyro errors are as follows: (1) choosing a motor with a smoother and steadier speed and (2) selecting inertial sensors with as little bias as possible.

Conversely, considering the existence of a non-orthogonal angle between the rotation axis and the radial sensitive axis, we defined the non-orthogonal angles as $\eta_{zx}$ and $\eta_{zy}$, respectively. According to Yong Jia [43], the introduced gyro equivalent constant drift can be expressed as follows:

(29) $$\delta \omega_{x}=(\eta_{zx}\cos\omega t-\eta_{zy}\sin\omega t)\omega$$

(30) $$\delta \omega_{y}=(\eta_{zx}\sin\omega t+\eta_{zy}\cos\omega t)\omega$$

After the installation of the MEMS IMU, the orthogonal angle is a constant value. If the rotational speed is stable, the integration of $\delta \omega_{x}$ and $\delta \omega_{y}$ within one rotation cycle are both approximately zero. However, the rotational speed usually fluctuates, which reduces the effect of RMT. Therefore, two measures to reduce the error caused by the non-orthogonal angle include: (1) improving the smoothness of the motor rotation speed and (2) reducing the non-orthogonal angle error and performing calibration compensation. The specific error compensation method was previously reported [43].

## 6. Conclusions

The RMT of the MEMS IMU has become a hot research topic, as it can substantially reduce the effect of the bias of inertial sensors. The main contribution of this paper is the proposal of a method for the self-alignment of the MEMS IMU under swing conditions based on the RMT-IFBA method. In this paper, the rotation scheme design and the algorithm principle were analyzed in detail. The effectiveness of the proposed method was theoretically analyzed using a simulation test, and then turntable experiments were conducted to corroborate the feasibility and reliability of the proposed method in practical systems. Based on the analysis and results, the following conclusions were drawn: (1) Using RMT-IFBA for coarse alignment and STF for fine alignment can successfully align and the MEMS IMU without requiring assistance from external information, which is the main innovation of this paper. The standard deviations of the pitch, roll, and heading were 0.0140°, 0.0097°, and 0.91°, respectively. (2) The RMT that MEMS IMU rotates around the *z*-axis is crucial to achieving the self-alignment of the MEMS IMU, which allows small misalignment angles during coarse alignment. (3) Both filtering methods, KF and STF, significantly improved the alignment accuracy, but STF was superior in tracking the changes in attitudes. (4) Each of the approaches covered in this paper were not new, but some new results were obtained through the joint use of these techniques. (5) Increasing the smoothness of the rotation rate and compensating for the non-orthogonal angle improved the effect of the RMT. (6) The inertial sensors used in the experiment were designed by our laboratory, with higher accuracy than conventional commercial MEMS inertial sensors. Therefore, with further improvement in the accuracy of MEMS inertial sensors, the same method could obtain even more accurate results. (7) The method presented in this paper provides a new idea for expanding the application range and improving the performance of the MEMS IMU. However, further investigations into alignment algorithms in more complex practical circumstances are required. 

To summarize, the results indicated that the proposed method can provide comparable accuracy when aligning the MEMS IMU. This method is reliable, and it is expected to be applied in small UAVs, small ships, indoor positioning, short-time navigation, and many other fields. More algorithms and better inertial sensors need to be explored further. An important future task is to design a practical rotary MEMS IMU.

## Figures and Tables

**Table 1:** The swing parameters.

Item	Pitch	Roll	Heading
Amplitude (°)	5	8	10
Cycle (s)	6	7	5
Swing center (°)	0	0	30

**Table 2:** Sensor errors.

Axes	Gyro error		Accelerometer Error	
	Constant Drift (°/h)	Random Noise (${}^{\circ}/\sqrt{h}$)	Constant Bias (μg)	Random Noise ($\mu g/\sqrt{Hz}$)
*x*-axis	10	0.02	100	10
*y*-axis	10	0.02	100	10
*z*-axis	10	0.02	100	10

**Table 3:** Alignment errors of the MEMS IMU rotating around the *z*-axis.

Statistical Parameters	Pitch Error (°)	Roll Error (°)	Heading Error (°)
Mean (°)	0.0189	–0.0351	0.2667
SD (°)	0.1130	0.0963	0.5475

**Table 4:** MEMS IMU specifications.

Parameter	Gyro	Accelerometer
Repetitiveness of bias	10°/h(1σ)	0.5 mg (1σ)
Random noise	$0.02{}^{\circ}/\sqrt{h}$	$5\;\mu g/\sqrt{Hz}$
Measuring range	±300°/s	±20 g

**Table 5:** Parameters of Scheme 1.

Items	Inner frame	Middle frame	Outer frame
Amplitude (°)	6	10	-
Frequency (Hz)	0.125	0.1	-
Rotation rate (°/s)	-	-	20

**Table 6:** Parameters of Scheme 2.

Item	Inner Frame	Middle Frame	Outer Frame
Amplitude (°)	-	6	10
Frequency (Hz)	-	0.125	0.1
Rotation rate (°/s)	20	-	-

**Table 7:** Standard deviation of the coarse alignment in Scheme 1.

Statistical Parameters	Pitch Angle (°)	Roll Angle (°)	Heading Angle (°)
Mean	−0.12	0.43	164.17
SD	0.03	0.02	1.49

**Table 8:** The mean and standard deviation of three attitude angles.

Filtering Method	Pitch (°)		Roll (°)		Heading (°)	
	Mean	SD	Mean	SD	Mean	SD
KF	0.0041	0.0183	0.0035	0.0139	166.29	1.20
STF	−0.0081	0.0140	−0.0041	0.0097	165.96	0.91
